# Supplementary material for: Immune-cancer analyses across mammals reveal a potential trophic level and platelet-linked tradeoff between cancer and trauma mortality
Source: bioRxiv. 2025 Dec 11:2025.12.09.693265. Preprint. [Version 1] doi: 10.64898/2025.12.09.693265 (PMC12822737; doi:10.64898/2025.12.09.693265)
Supplement: Supplementary file 1 [file NIHPP2025.12.09.693265v1-supplement-4.pdf]

bioRxiv preprint doi: https://doi.org/10.64898/2025.12.09.693265; this version posted December 11, 2025. The copyright holder for this preprint (which was not certified by peer review) is the author/funder, who has granted bioRxiv a license to display the preprint in perpetuity. It is made available under a CC-BY 4.0 International license.

Supplementary Material

Supplementary Information

In our dataset of 216 mammalian species across 18 different taxonomic orders, we found that different taxonomic orders have different median concentrations of white blood cells (Supp. Fig. 14A; no statistically significant outliers), lymphocytes (Supp. Fig. 14B; with 2 statistically significant outliers: the Egyptian fruit bat *Rousettus aegyptiacus* and the Linnaeus's two-toed sloth *Choloepus didactylus*), monocytes (Supp. Fig. 14E; with 2 statistically significant outliers: the Egyptian fruit bat *Rousettus aegyptiacus* and the Celebes crested macaque *Macaca nigra*), platelet size (Supp. Fig. 14F; no statistically significant outliers), platelet concentration (Supp. Fig. 14G; no statistically significant outliers), cancer mortality (Supp. Fig. 14H; with 8 statistically significant outliers: the European mink *Mustela lutreola*, the European hamster *Cricetus cricetus*, the Tasmanian devil *Sarcophilus harrisii*, the gray mouse lemur *Microcebus murinus*, the European polecat *Mustela putorius*, the brown rat *Rattus norvegicus*, the four-toed hedgehog *Atelerix albiventris*, and the kowari *Dasyuroides byrnei*), and trauma mortality (Supp. Fig. 14I; no statistically significant outliers). However, the concentration of segmented neutrophils (Supp. Fig. 14C; with one statistically significant outlier: the pygmy marmoset *Cebuella pygmaea*) or the segmented neutrophil-to-lymphocyte ratio (Supp. Fig. 14D) does not differ among the examined taxonomic orders.

The reason for the very high concentration of immune cells in outlier species is largely unknown. There were two outlier species with relatively high lymphocyte concentrations in our dataset, the Egyptian fruit bat *Rousettus aegyptiacus* and the Linnaeus's

bioRxiv preprint doi: https://doi.org/10.64898/2025.12.09.693265; this version posted December 11, 2025. The copyright holder for this preprint (which was not certified by peer review) is the author/funder, who has granted bioRxiv a license to display the preprint in perpetuity. It is made available under a CC-BY 4.0 International license.

two-toed sloth *Choloepus didactylus*. Vogel et al.[120] found a similar mean value of 5.0 x 10^9 cells/L lymphocytes from 66 wild-caught healthy individual Linnaeus's two-toed sloths (*Choloepus didactylus*) to the median value of 5.78 x 10^9 cells/L lymphocytes from 94 healthy individual Linnaeus's two-toed sloths (*Choloepus didactylus*) under human care[121] in our study. However, the reason for the relatively high lymphocyte concentrations in these species is unknown. The Egyptian fruit bat (*Rousettus aegyptiacus*) was also an outlier species with very high (9.2 x 10^9 cells/L; from 26 different individuals; both healthy male and female Egyptian fruit bats *Rousettus aegyptiacus* in ZIMS of unspecified animal age[122]) monocyte concentrations, whereas previous literature in 3 mock-inoculated juvenile male Egyptian fruit bats (*Rousettus aegyptiacus*) has reported monocyte concentrations lower than 2 x 10^9 cells/L[123]. Celebes crested macaques (*Macaca nigra*), are an outlier species with very high monocyte concentrations; a median across 203 healthy individuals[124]. There is no reference value of monocyte counts in previous literature for this species and no clear explanation as to why this species has such high monocyte concentrations. Similarly in the case of the pygmy marmoset (*Cebuella pygmaea*), an outlier with relatively high concentrations of segmented neutrophils across 65 healthy individuals, there is no previous literature on reference counts of segmented neutrophils for this species or a clear explanation for its high segmented neutrophil concentrations. The way that these animals were handled for blood collection, potentially anesthesia or manual restraint, may have affected the concentrations of these immune cells[97]. In the ZIMS database, consistent data entry errors, confusion over test methodology, or unit of measurement conversations over time by different individuals entering the data may also have an impact on data quality.

Regarding immune profiles and body mass, our comparative analyses showed distinct immune profiles in larger versus smaller species but no indication of which immune profiles are better at preventing mortality from cancer cells, ‘non-self’ cells, and/or autoimmune

bioRxiv preprint doi: https://doi.org/10.64898/2025.12.09.693265; this version posted December 11, 2025. The copyright holder for this preprint (which was not certified by peer review) is the author/funder, who has granted bioRxiv a license to display the preprint in perpetuity. It is made available under a CC-BY 4.0 International license.

diseases. Our findings of higher segmented neutrophil-to-lymphocytes ratios and lower lymphocyte concentrations in larger species align with previous findings in some taxa. Downs et al.[21] and Naidenko and Alshinetskiy[24] also used the ZIMS database to collect species' immune cell data but from a different time period (which explains why their values may not be exactly the same as ours). Downs et al.[21] found that body mass is positively correlated with neutrophil concentrations across 259 terrestrial mammalian species, and Naidenko and Alshinetskiy found that body mass is negatively correlated with lymphocyte concentration across 25 felids (*P*-value = 0.0094)[24]. Without measures of immune cell function across species it is difficult to understand why and how these correlations exist, and which immune profiles are indicative of ability to prevent death from different threats.

All *P*-values in the article are un-adjusted for multiple testing corrections.

Supplementary Movie. Figure 6 in the form of a movie. Variables that have the same font size at the same time are positively correlated (a trend: no line joining them; statistical significance after FDR correction: a line joining them). Whereas when one variable has a large font size and the other has a small font size at the same time, this represents that they are negative correlated (a trend: no line joining them; statistical significance after FDR correction: a line joining them).

Supplementary Data. The species-level life history, immune cell, cancer prevalence, cancer mortality, and trauma mortality data used in this study. We will share these data upon acceptance of the manuscript for publication.

Supplementary Figure 1. Body mass (A) and lifespan (B) are not correlated with the median concentration of white blood cells. The analysis in A is controlled for lifespan, whereas the analysis in B is controlled for body mass. Each dot shows the median concentration of white blood cells and body mass (A) or the lifespan (B) of one species. The

bioRxiv preprint doi: https://doi.org/10.64898/2025.12.09.693265; this version posted December 11, 2025. The copyright holder for this preprint (which was not certified by peer review) is the author/funder, who has granted bioRxiv a license to display the preprint in perpetuity. It is made available under a CC-BY 4.0 International license.

regression lines show the phylogenetically-controlled linear regression of body mass (A) and lifespan (B) versus the median concentration of white blood cells.

Supplementary Figure 2. Body mass (A) and lifespan (B) are not correlated with the median concentration of segmented neutrophils. The analysis in A is controlled for lifespan, whereas the analysis in B is controlled for body mass. Each dot shows the median concentration of segmented neutrophils and body mass (A) or the lifespan (B) of one species. The regression lines show the phylogenetically-controlled linear regression of body mass (A) and lifespan (B) versus the median concentration of segmented neutrophils.

Supplementary Figure 3. Body mass (A), but not lifespan (B), is negatively correlated with the median concentration of lymphocytes. The analysis in A is controlled for lifespan, whereas the analysis in B is controlled for body mass. Each dot shows the x and y variables of one species. The regression lines show the phylogenetically-controlled linear regression of body mass (A) and lifespan (B) versus the median concentration of lymphocytes.

Supplementary Figure 4. Body mass (A), but not lifespan (B), is positively correlated with the ratio of segmented neutrophils-to-lymphocytes. Brackets indicate concentrations. The analysis in A is controlled for lifespan, whereas the analysis in B is controlled for body mass. Each dot shows the x and y variables of one species. The regression lines show the phylogenetically-controlled linear regression of body mass (A) and lifespan (B) versus the ratio of segmented neutrophils-to-lymphocytes.

Supplementary Figure 5. Body mass (A) and lifespan (B) are not correlated with the median concentration of monocytes. The analysis in A is controlled for lifespan, whereas the analysis in B is controlled for body mass. Each dot shows the median concentration of monocytes and body mass (A) or lifespan (B) of one species. The regression lines show the phylogenetically-controlled linear regression of body mass (A) and lifespan (B) versus the median concentration of monocytes.

bioRxiv preprint doi: https://doi.org/10.64898/2025.12.09.693265; this version posted December 11, 2025. The copyright holder for this preprint (which was not certified by peer review) is the author/funder, who has granted bioRxiv a license to display the preprint in perpetuity. It is made available under a CC-BY 4.0 International license.

Supplementary Figure 6. Body mass (A) and lifespan (B) are not correlated with median platelet size. The analysis in A is controlled for lifespan, whereas the analysis in B is controlled for body mass. Each dot shows median platelet size and body mass (A) or lifespan (B) of one species. The regression lines show the phylogenetically-controlled linear regression of body mass (A) and lifespan (B) versus median platelet size.

Supplementary Figure 7. Body mass (A) and lifespan (B) are not correlated with median platelet concentration. The analysis in A is controlled for lifespan, whereas the analysis in B is controlled for body mass. Each dot shows median platelet concentration and body mass (A) or lifespan (B) of one species. The regression lines show the phylogenetically-controlled linear regression of body mass (A) and lifespan (B) versus median platelet concentration.

Supplementary Figure 8. The median concentration of segmented neutrophils is not correlated with cancer prevalence (A) nor cancer mortality (B). Each dot shows the cancer prevalence (A) or cancer mortality (B) and the median concentration of segmented neutrophils of one species. The regression lines show the correlation between the median concentration of segmented neutrophils and cancer prevalence (A) or cancer mortality (B).

Supplementary Figure 9. The ratio of segmented neutrophils-to-lymphocytes is not correlated with cancer prevalence (A) nor cancer mortality (B). Brackets indicate concentrations. Each dot shows the cancer prevalence (A) or cancer mortality (B) and the ratio of segmented neutrophils-to-lymphocytes of one species. The regression lines show the correlation between the ratio of segmented neutrophils-to-lymphocytes and cancer prevalence (A) or cancer mortality (B).

Supplementary Figure 10. The median concentration of lymphocytes is not correlated with cancer prevalence (A) nor with cancer mortality (B). Each dot shows the cancer prevalence (A) or cancer mortality (B) and the median concentration of lymphocytes of one

bioRxiv preprint doi: https://doi.org/10.64898/2025.12.09.693265; this version posted December 11, 2025. The copyright holder for this preprint (which was not certified by peer review) is the author/funder, who has granted bioRxiv a license to display the preprint in perpetuity. It is made available under a CC-BY 4.0 International license.

species. The regression lines show the correlation between the median concentration of lymphocytes and cancer prevalence (A) or cancer mortality (B).

Supplementary Figure 11. The median concentration of monocytes is not correlated with cancer prevalence (A) nor cancer mortality (B). Each dot shows the cancer prevalence (A) or cancer mortality (B) and the median concentration of monocytes of one species. The regression lines show the correlation between the median concentration of monocytes and cancer prevalence (A) or cancer mortality (B).

Supplementary Figure 12. The median concentration of white blood cells is not correlated with cancer prevalence (A) nor cancer mortality (B). Each dot shows the cancer prevalence (A) or cancer mortality (B) and the median concentration of white blood cells of one species. The regression lines show the correlation between the median concentration of white blood cells and cancer prevalence (A) or cancer mortality (B).

Supplementary Figure 13. Correlations between platelet size and concentration, cancer prevalence, cancer mortality, trauma mortality, and lifespan. There are no correlations between the examined variables (A–E). Each dot shows the x and y variables of one species. Each regression line shows the phylogenetically-controlled linear regression between the independent and dependent variable.

Supplementary Figure 14. Immune profiles, cancer mortality, and trauma mortality across different mammalian taxonomic orders. The median concentration of white blood cells (A), monocytes (E), platelet size (F), platelet concentration (G), cancer mortality (H), and trauma mortality (I) are different among orders. Each dot shows the x and y variables of one species. The horizontal line in each violin shows the median value of the y variable in the taxonomic order shown on the x axis.

bioRxiv preprint doi: https://doi.org/10.64898/2025.12.09.693265; this version posted December 11, 2025. The copyright holder for this preprint (which was not certified by peer review) is the author/funder, who has granted bioRxiv a license to display the preprint in perpetuity. It is made available under a CC-BY 4.0 International license.

Supplementary Figure 15. The sum of trauma and cancer mortality percentages across the examined species follow a normal distribution (211 species, Shapiro's test: *P*-value = 0.61). Each point is a species.

Supplementary Figure 16. When excluding the Felidae, cancer prevalence remains negatively correlated with platelet concentration. Each dot shows the median platelet concentration and cancer prevalence of one species. The regression line shows the phylogenetically-controlled linear regression of median platelet concentration versus cancer prevalence.

Supplementary Figure 17. There is a non-statistically significant trend for higher trophic levels to have larger platelets. Each dot shows the median platelet concentration and cancer prevalence of one species. The horizontal line in each violin shows the median value of the y variable in the taxonomic order shown on the x axis. Herbivores do not have smaller platelets than the other trophic levels ($P > 0.05$; Table 1).

Supplementary Figure 18. Median platelet size is negatively correlated with median platelet concentration. Each dot shows the median platelet size and the median platelet concentration of one species. The regression line shows the correlation between median platelet size and median platelet concentration.

Supplementary Figure 19. Cancer prevalence is positively correlated with cancer mortality. Each dot shows the cancer prevalence and the cancer mortality of one species. The regression line shows the correlation between cancer prevalence and cancer mortality.

bioRxiv preprint doi: https://doi.org/10.64898/2025.12.09.693265; this version posted December 11, 2025. The copyright holder for this preprint (which was not certified by peer review) is the author/funder, who has granted bioRxiv a license to display the preprint in perpetuity. It is made available under a CC-BY 4.0 International license.

Table 1

Summary phylogenetic regression (PGLS) statistics. We note with an asterisk (*) on the *P*-value and **in bold the regressions that are considered statistically significant after the False Discovery Rate corrections**. High lambda values show that the associations are mainly explained by common ancestry. Multivariable additive PGLS models are shown as rows with multiple independent variables but only one dependent variable. In the cases where trophic level is the independent variable, herbivores are the reference category. Prevalence and mortality data are on the unit interval and many values were near zero, so these data are arcsine-square root transformed for the regression analyses.
